# Identification of Acute Malnutrition in Children in Cambodia Requires Both Mid Upper Arm Circumference and Weight-For-Height to Offset Gender Bias of Each Indicator

**DOI:** 10.3390/nu10060786

**Published:** 2018-06-19

**Authors:** Frank Tammo Wieringa, Ludovic Gauthier, Valérie Greffeuille, Somphos Vicheth Som, Marjoleine Amma Dijkhuizen, Arnaud Laillou, Chhoun Chamnan, Jacques Berger, Etienne Poirot

**Affiliations:** 1 UMR-204 Nutripass, Institut de Recherche pour le Développement, IRD/UM/SupAgro, 34390 Montpellier, France; valerie.greffeuille@ird.fr (V.G.); jacques.berger@ird.fr (J.B.); 2 UNICEF-Cambodia, Phnom Penh, Cambodia; gauthier.ludo@hotmail.fr (L.G.); alaillou@unicef.org (A.L.); epoirot@unicef.org (E.P.); 3 Department of Health Sciences, Vrije Universiteit Amsterdam, 1081 HV Amsterdam, The Netherlands; somphosvichethsom@gmail.com; 4 Department of Nutrition, Excercise and Sports, Copenhagen University, 1165 Copenhagen, Denmark; madijkhuizen@gmail.com; 5 Department of Fisheries Post-Harvest Technologies and Quality Control, Fisheries Administration, Ministry of Agriculture, Forestry and Fisheries, Phnom Penh, Cambodia; chhounchamnan@gmail.com

**Keywords:** malnutrition, anthropometry, weight-for-height, mid upper arm circumerference (MUAC), gender

## Abstract

Malnutrition remains a serious health problem in Cambodia with over 10% of children less than five years of age suffering from acute malnutrition. In addition to the presence of nutritional edema, two indicators are recommended by the World Health Organization for the diagnosis of acute malnutrition: weight-for-height Z-scores (WHZ; with acute malnutrition defined as WHZ < −2 Z-score) and mid-upper arm circumference (MUAC, with acute malnutrition defined as MUAC < 12.5 cm). Earlier, we showed that WHZ and MUAC identified different subgroups of children with acute malnutrition. To explore factors associated with both indicators of acute malnutrition, we analyzed baseline data from a longitudinal study in three provinces in Cambodia: Phnom Penh (capital, urban environment), Kratie (rural province), and Ratanakiri (hilly, rural province). Data was available for 4381 children below 30 months of age. Malnutrition rates were higher in the two rural provinces than in the capital. Although both MUAC and WHZ showed gender bias, with MUAC identifying more girls, and WHZ identifying more boys with acute malnutrition, the gender effect was strongest for MUAC. The gender bias of MUAC diminished with older age, but remained significant up to 30 months of age. Only using both MUAC and WHZ as indicators resulted in gender neutral identification of acute malnutrition. WHZ alone always identified more children with acute malnutrition than MUAC alone. In Phnom Penh, MUAC alone identified only 11% with acute malnutrition in addition to WHZ. To conclude, both MUAC and WHZ showed gender bias in this cohort of Cambodian children. In Cambodia, implementation of a MUAC-only or a WHZ-only program for the identification of acute malnutrition would be unethical as it will lead to many children remaining undiagnosed.

## 1. Introduction

Acute malnutrition (AM), which includes both marasmus and kwashiorkor, is still a major health threat in children, associated with a remarkably higher risk for mortality and morbidity. Children who are wasted have an almost five times higher risk to die, a risk that increases to over 12 times when stunting is also present [1]. An estimated 19 million children are affected by severe acute malnutrition every year, and an estimated 34 million suffer from moderate acute malnutrition [2]. Therefore, screening children for acute malnutrition is an essential component of preventive health care programs in most countries [3].

In addition to the presence of nutritional edema, indicating kwashiorkor, the World Health Organization recommends using either weight-for-height Z scores (WHZ) and/or mid-upper arm circumference (MUAC) for diagnosing acute malnutrition in children above six months of age [4]. Whereas WHZ has been used for several decades in the diagnosis of nutritional status in children, MUAC was introduced in the 1990s as an easier tool to use in field settings [5], and appeared to be a more precise indicator to predict mortality in malnourished children [6]. However, in South-Sudan, MUAC < 11.5 cm failed to identify 1/3 of the children who died of acute malnutrition while admitted to a therapeutic feeding program [7]. A child above six months of age is considered being wasted if WHZ < −2 and/or MUAC < 12.5 cm. Acute malnutrition is further distinguished as being severe (WHZ < −3 scores and/or MUAC < 11.5 cm) or moderate (−3 < WHZ < −2 and/or 11.5 < MUAC < 12.5). For infants <six months of age, the same WHZ-scores are used to identify acute malnutrition. Cut-offs for MUAC in infants <six months have not been established but some researchers have argued that MUAC is a more reliable indicator than WHZ in infants <six months of age [8].

However, as recently shown by us, MUAC and WHZ identified two different subgroups of Cambodian children suffering from malnutrition [9], with little overlap between them. This is not a unique feature of Cambodian children. Grellety and Golden reported data on WHZ and MUAC from 47 countries [10]. In none of these countries did MUAC and WHZ identify more than 40% of the same children as being wasted. On average, both criteria were concordant in only 27%, or roughly in one out of four children with acute malnutrition [10]. Using only one criteria (either MUAC or WHZ) will, therefore, result in the majority of children with acute malnutrition not receiving the warranted treatment.

Briend and colleagues have argued that genetic differences in fat distribution, for example, the different fat distribution in Asian populations, or differences in length or height could explain the differences between MUAC and WHZ to identify acute malnutrition [11]. To identify possible factors associated with either a low MUAC or a low WHZ-score, we analyzed anthropometrical data of young children, participating in a large longitudinal survey, the MyHealth project, in Cambodia.

## 2. Methods

The MyHealth study is a longitudinal cohort study, conducted in three provinces in Cambodia: Phnom Penh (the capital, urban environment), Kratie (around 200 km to the northeast of Phnom Penh, mainly rural environment surrounding the Mekong River) and Ratanakiri (400 km to the northeast of Phnom Penh, hilly rural environment). For the cohort study over 1000 children and 1000 women were recruited in each province.

Lists of pregnant and lactating women, women in reproductive age (WRA) and children under three years were obtained from village health volunteers (VHSGs) and midwives in selected operational districts. In total 5419 households were recruited and teams visited each household to collect data on socio-economic data, dietary intake, water, sanitation and hygiene, and anthropometry of mother and child. In addition, in each province, pregnant women were recruited into the study also, but these are not considered in the present study. Eligible children were children aged between 0 and 30 months of age, and not suffering from a chronic health condition requiring medication [12]. Data for the baseline of the cohort study was collected by an experienced and trained team.

Children’s heights were measured to the nearest 1 mm. Height measurements (UNICEF measurement boards) were collected in triplicate from each child in the household under three years to ensure accuracy. Weight were measured to the nearest 0.1 kg (Seca; Hamburg, Germany). The nutritional status of children was defined length or height-for-age (L/HAZ), weight-for-height (WHZ), and weight-for-age (WAZ) z-scores, calculated according to the Child Growth Standard of the World Health Organization (WHO) using the WHO Anthro software. To ensure the accuracy of the data, extreme values were excluded from the analysis: WAZ < −6 or > 5; L/HAZ < −6 or >5; WHZ < −6 or > 5. Excluded values represented less than 5% of the total values. We used the following cut-offs to define wasting: weight for height < −2 Z-scores (according to the WHO growth charts) or MUAC < 12.5 cm, and to define severe acute malnutrition (SAM): weight for height < −3 Z-scores or MUAC < 11.5 cm. Stunting was defined as a height-for-age < −2 Z-scores.

Ethical approval for the MyHealth study was obtained from the Cambodia National Ethical Committee for Health Research, National Institute of Public Health, Ministry of Health, Cambodia. Informed consent was obtained from all participants, with consent obtained from parents or guardians for participating children. All data collected is anonymized and stored on the Kobotoolbox server, with access to the data limited to the core team of researchers only.

## 3. Data Collection and Statistical Analysis

Data was collected on tablets, using the Koboltoolbox software (www.kobotoolbox.org). Anthropometrical indicators (weight-for-age, height-for-age, weight-for-height) were calculated according to the WHO growth standards [13]. Data was checked for outliers and improbable values were deleted (all Z-score < −6 or > +5). To study the within-subject concordance of acute malnutrition diagnosed by WHZ vs diagnosed by MUAC, we categorized individuals in four categories: subjects with a WHZ < −2 Z-score, but a MUAC ≥ 12.5 cm; subjects with a MUAC < 12.5 cm, but a WHZ ≥ −2 Z-score; subjects with both MUAC<12.5 cm and WHZ < −2 Z-score and non-wasted subjects, defined as individuals with both WHZ > −2 Z-score and MUAC > 12.5 cm. This four-category variable was used as the response variable in a multinomial regression model to assess the relationship between the two diagnostics and the selected covariates: place of residence, socio-economic position (wealth index and mother education), and physiological factors (gender of the child, age in months, and whether the child is stunted or not). The non-wasted children category was used as a reference and, consequently, the relative risk ratio (RRR) of each category is computed relative to non-wasted children. For each variable included in the model, both univariate and adjusted coefficients are presented.

To explore the relationship between age and the general acute malnutrition indicators, we fitted a multinomial logit model for every region using a fractional polynomial for the age variable. Probabilities for each diagnosis were then estimated and plotted for the 6–30 month age range. The same procedure was done to assess the relationship with height-for-age z-score.

We also investigated potential gender bias of MUAC and WHZ as indicators for acute malnutrition. For each indicator, a new variable with three categories was created, recoding the four category variables described above. Thus, to analyze gender bias of MUAC diagnosis, children were categorized as: those with a MUAC < 12.5 cm; children with a WHZ < −2, but a MUAC ≥ 12.5 cm; and non-wasted children (WHZ > −2 and MUAC > 12.5 cm), used as reference category. For the WHZ diagnosis, subjects were categorized in a similar way: children with a WHZ < −2 cm; children with a MUAC < 12.5 cm, but a WHZ ≥ −2; and non-wasted children (WHZ > −2 and MUAC > 12.5 cm). We then performed two multinomial logistic regression models using these categorical variables as a response, adjusting for all the selected covariates, and then plotted the average marginal effect of the gender variable on the indicator category for several values of age. To examine if there was any gender bias when one considers the two diagnoses together (i.e., MUAC < 12.5 cm and/or WHZ < −2), we created a binary variable taking the value 0 if the child is not wasted and 1 if s/he is diagnosed by any of the two indicators. This variable was then used as a response variable in a logistic model including all the covariates, and the average marginal effect of the gender variable was estimated.

We used Stata 13 for data management and Stata 13 (Stata Corp., College Station, TX, USA) and R software version 3.4.0 for analysis. The type I error risk was set to 0.05 and the complete cases analysis was used to deal with missing data for all the multivariate analyses.

## 4. Results

Data were available for 4381 children (1494 from Phnom Penh, 1533 from Kratie, and 1354 from Ratanakiri, Table 1). There was no case of edematous malnutrition. The overall prevalence of AM (defined by WHZ < −2 and/or a MUAC < 12.5 cm) was significantly lower in urban Phnom Penh (12.2%) than in the two rural sites (*P* < 0.05, chi-square). Overall prevalence of AM was lower in Ratanakiri (17.2%) than in Kratie (23.5%, *P* < 0.05).

When using only MUAC to diagnose acute malnutrition, and restricting the analyses to infants older than six months, the prevalence of acute malnutrition for Phnom Penh became only 4.2%, that is 47 children were identified as having acute malnutrition, out of 141 (12.5%) when using both WHZ and MUAC as an indicator. The remaining 42 children with AM in Phnom Penh were below six months of age, and would not have been identified with MUAC, as MUAC is used only in children above six months of age. Hence, in Phnom Penh, only 1/3 of the malnourished children above six months of age was identified with MUAC alone. For Kratie and Ratanakiri, MUAC alone identified 146 and 136 children, respectively (47.6% and 62.1% of all children with AM, respectively). In contrast, in children above six months of age, WHZ alone identified 121 (85.8%), 250 (81.4%), and 161 (73.5%) in Phnom Penh, Kratie and Ratanakiri, respectively.

To compare factors associated with MUAC and/or WHZ, we have restricted the further analysis to children older than six months of age. Factors associated with having only a low MUAC included younger age, being female (aRRR 3.2; 95%CI 2.1–5.0), being stunted (aRRR 4.9; 95%CI 3.3–7.3) or living in a rural province (aRRR 2.3 and 2.0 for Kratie and Ratanakiri, respectively, *P* = 0.021, Table 2). In contrast, factors associated with having only a WHZ < −2 included being older (*P* < 0.01), being stunted (aRRR 1.9; 95%CI 1.5–2.5) and being male (aRRR 1.9; 95%CI 1.5–2.5, Table 2). When using WHZ only, the differences between the urban site and rural sites became less apparent, with only living in Kratie being a risk factor for a low WHZ score. When combining the two indicators for malnutrition, the age and gender effects were attenuated, but still statistical significant.

**Figure 1:**
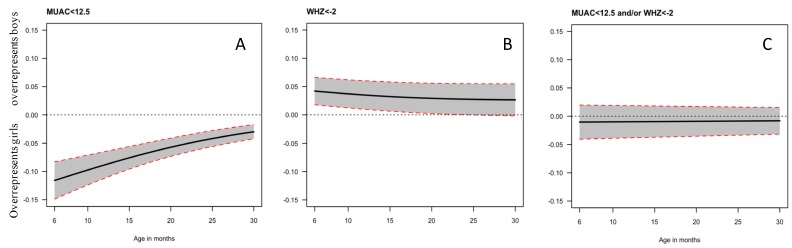
Effect of gender on the probability of being diagnosed with acute malnutrition when using mid-upper arm circumference (MUAC) only (**A**), weight-for-height Z-score (WHZ) only (**B**) or both indicators (**C**). An overrepresentation of girls is reflected by a curve below zero, whereas an overrepresentation of boys is reflected by a curve above zero.

The gender bias of MUAC and WHZ as indicator for acute malnutrition is reflected in the figures (Figure 1A–C) below. In our dataset, using MUAC as indicator for malnutrition (Figure 1A) gives a 10% higher absolute probability for girls to be diagnosed with AM than boys. The effect is strongest at six months of age, with a >10% difference in probability between girls and boys being diagnosed with AM, but the bias remains significant up to 30 months of age. In contrast, boys have a higher probability of being diagnosed with AM when using WHZ as an indicator than girls (Figure 1B), with about a 4% higher probability, which is no longer statistically significant after 20 months of age. When combining MUAC and WHZ (Figure 1C), there is no longer any gender bias.

**Figure 2:**
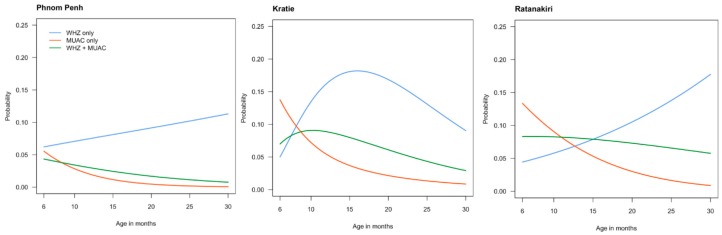
Prevalence of wasting in the three study sites, when using WHZ alone (<−2 Z-score) or MUAC alone (<12.5 cm). The group WHZ + MUAC consists of children having both a WHZ < −2 Z scores and a MUAC < 12.5 cm.

To explore the age relationship between acute malnutrition and the two indicators for acute malnutrition, we plotted the probability to be diagnosed with AM by the two indicators alone or combined. As can be seen in Figure 2, the patterns of the two indicators for AM differed considerably between the three sites. In all three sites, MUAC alone became less important as an indicator for AM with increasing age.

In urban Phnom Penh, WHZ alone always diagnosed more children with AM than MUAC alone, whereas in the two rural study sites, MUAC alone diagnosed most of the infants with acute malnutrition between 6 and 12 months of age, before rapidly becoming less important. In urban Phnom Penh and rural Ratanakiri, there was a steady increase in the prevalence of acute malnutrition between 6 and 30 months of age, which was almost exclusively driven by an increase in the number of children having a WHZ < −2. In contrast, in rural Kratie, the prevalence of AM picked around 18 months of age. For all sites, after 18 months of age, WHZ alone became by far the most important indicator to diagnose acute malnutrition with >50% of the cases being diagnosed through WHZ alone.

**Figure 3:**
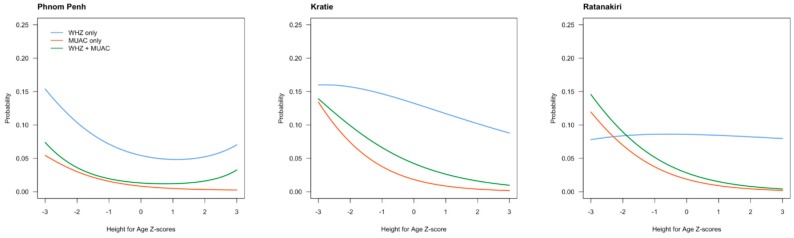
Probability of being diagnosed with acute malnutrition using WHZ scores, MUAC, or both combined as an indicator, in relation to height-for-age Z score (HAZ) in children living in Phnom Penh, Kratie, or Ratanakiri.

To further explore the relationship between height and indicators of acute malnutrition, we made similar curves for HAZ scores (Figure 3).

In general, there was a negative association between the probability of being diagnosed with AM and HAZ, that is, being taller reduces the likelihood for having AM. MUAC as indicator for AM tended to be more affected by length status than WHZ. However, only in Ratanakiri was WHZ as an indicator for AM not related to the HAZ score at all.

## 5. Discussion

The present study confirms the earlier study from Cambodia that MUAC and WHZ identify different sub-sets of children [9] and it highlights the importance of including both indicators for the diagnosis of AM. The gender bias of MUAC, with more girls than boys being identified with AM, has been described before [14]. However, in our dataset, both indicators for the diagnosis of AM were gender-dependent, although to a much larger extent for MUAC than WHZ. While a low MUAC was found more often in girls, a low WHZ was more prevalent in boys. It is important to point out that while there are different WHZ curves for boys and girls, no gender-specific cut-off is applied for MUAC. Hence, the gender bias of MUAC might partly be remedied by introducing gender-specific cut-offs. However, earlier we reported that differences in optimal cut-offs for MUAC to identify a low WHZ were no more than 0.5 cm [14], making gender-specific cut-offs for MUAC perhaps impractical.

The present study has some important practical implications. It has been argued that children with a low MUAC are at an especially higher risk of death [6], and that for cost-benefit reasons, MUAC-only programs should be implemented [15]. However, as argued before, younger children are always at a higher risk for mortality than older children, and the higher mortality risk associated with MUAC might just reflect the age (and gender) bias of MUAC as an indicator. In the present study, in urban Phnom Penh, most children with AM would have been identified with WHZ alone, as MUAC alone added only 20 additional cases of AM (10.9%). In contrast, a MUAC-alone program would have identified only 47 cases of AM, leaving 74.3% of the cases of AM unidentified, a clearly unethical situation. Hence, for Cambodia, changing the current program which uses WHZ to a MUAC-alone program is not desirable. Another issue are infants younger than six months of age. MUAC is currently only used for children above six months of age, but in our cohort, there were 109 infants (out of 1114 infants, 9.8%) below six months of age diagnosed with AM by WHZ, which would not have been picked up by a MUAC-alone program.

Another important policy implication of the current study is that unfortunately, programs using WHZ alone, are not suitable either. In Ratanakiri, MUAC alone identified 58 additional cases of AM, or 24.9% of the total. Hence, using a WHZ-alone program would have left almost 25% of the children with wasting unidentified. Earlier, we proposed the use of different cut-offs for MUAC for screening for AM, and for initiating treatment for AM [9]. Using a higher cut-off for MUAC for screening would result in more children with a WHZ < −2 to be identified. However, the downside of this approach is the higher burden to an already overwhelmed health system, with more children being referred to the health centers, many of them being sent home again, as they are not wasted. Therefore, to ensure that all children with wasting are identified, both MUAC and WHZ need to be used in the Cambodian setting to screen for acute malnutrition. It is also likely that this applies for many more countries.

In our current study, almost 18% of the children was identified as being wasted, with around 3% of SAM. In Cambodia, and in most other countries, no program exists for the treatment of MAM, and only cases of SAM are being treated. Indeed, it has been proven difficult to reach most children with SAM, with <10% of the estimated 60,000 SAM cases receiving treatment in 2014. However, an integrated approach, trying to improve the nutritional status of all children with AM, with standard treatment for children with SAM, and supplementary foods for children with MAM, might increase awareness in the Cambodian population for this serious health problem.

In the present study, and as reported earlier as well, a low MUAC was also associated with younger age and being stunted [9,14]. It is not surprising that MUAC is associated with age, as a single cut-off to define acute malnutrition is used for children between six months and five years of age, while MUAC is known to increase with age. Indeed, age-dependent cut-offs would be more appropriate [14], but have not been introduced due to logistic problems in the field. The hypothesis that WHZ as indicator for acute malnutrition tends to select tall, slender individuals is not supported by our data.

A weakness of the current study is that only cross-sectional data was available, with no long-term follow-up on subsequent risk for death or morbidity. As noted above, MUAC has been preferred above WHZ as an indicator for malnutrition, as MUAC was associated with a higher risk for death than a low WHZ [6]. However, in the present study, even if the risk for death in children with a low WHZ was only half of the risk in children with a low MUAC, still more children would have been identified, and potentially had received treatment, with a WHZ-alone program, as 63.5% of the children were identified by WHZ alone.

To conclude, the present study showed large within country differences in a relative small country as Cambodia, in the ability of MUAC and WHZ to identify children with acute malnutrition. Both indicators showed gender bias, but MUAC did to a far larger extent than WHZ. Overall, WHZ identified more children with acute malnutrition than MUAC. Linear growth status affected MUAC more than WHZ but, even in stunted children, WHZ identified more children overall with acute malnutrition than MUAC. To ensure that no child with acute malnutrition is left without proper treatment and follow-up, both MUAC and WHZ should be used as indicators for nutritional status in children < 5 years of age.

## Figures and Tables

**Table 1:** General characteristics of the study population, by province.

	Phnom Penh	Kratie	Ratanakiri	Overall	*P* ^1^
*N*	1494	1533	1354	4381	
Boys, *n* (%)	786 (52.6)	757 (49.4)	670 (49.5)	2213 (50.5)	NS
Age, mo (±SD)	11.9 (±6.8)	11.8 (±6.9)	12.0 (±7.2)	11.9	NS
HAZ (±SD)	−0.64 (±1.41) ^a^	−0.94 (±1.30) ^b^	−1.31 (±1.31) ^c^	−0.95 (±1.37)	<0.001
Stunted (%)	12.9 ^a^	18.1 ^b^	28.9 ^c^	19.7	<0.001
WAZ (±SD)	−0.80 (±1.14) ^a^	−1.32 (±1.11) ^b^	−1.37 (±1.12) ^b^	−1.15 (±1.15)	<0.001
Underweight (%)	13.3 ^a^	26.4 ^b^	28.0 ^b^	22.4	<0.001
WHZ (±SD)	−0.61 (±1.12) ^a^	−1.10 (±1.11) ^b^	−0.84 (±1.08) ^c^	−0.85 (±1.12)	<0.001
Wasted (<−2), *n* (%)	163 (10.9) ^a^	305 (19.8) ^b^	175 (12.9) ^a^	14.6	<0.001
SAM (<−3), *n* (%)	30 (2.0) ^a^	63 (4.1) ^b^	17 (1.3) ^a^	110 (2.5)	<0.001
*N* ^2^	1124	1151	992	3267	
MUAC cm (±SD)	14.3 (±1.2) ^a^	13.6 (±1.0) ^b^	13.5 (±1.2) ^b^	13.8 (±1.2)	<0.001
<12.5 cm, *n* (%)	47 (4.2) ^a^	146 (12.7) ^b^	136 (13.7) ^b^	10.1	<0.001
<11.5 cm, *n* (%)	6 (0.5) ^a^	19 (1.7) ^b^	15 (1.5) ^a,b^	40 (1.2)	0.033
AM ^3^, *n* (%)	183 (12.2) ^a^	360 (23.5) ^b^	233 (17.2) ^c^	776 (17.7)	<0.001

Abbreviations used: SD = standard deviation; HAX = height-for-age Z-score; WAZ = weight-for-age Z-score; WHZ = weight-for-height Z-score; MUAC = mid upper arm circumference; (S)AM = (severe) acute malnutrition. ^1^ *P*-Value for difference between provinces (ANOVA or chi-square). Rows with different superscript differ significantly from each other; ^2^ MUAC values are only for children older than six months of age; ^3^ AM was defined as either a WHZ < −2 Z score for all children and/or a MUAC < 12.5 cm for children above six months of age.

**Table 2:** Multinomial regression: wasting by MUAC only, WHZ only and both MUAC and WHZ compared to the non-wasted children; by place of residence, socio-economic and physiological factors.

		WHZ only (MUAC ≥ 12.5 cm & WHZ < −2) ^a^			Muac only (MUAC < 12.5 cm & WHZ ≥ −2) ^a^			MUAC and WHZ (MUAC < 12.5 cm & WHZ < −2) ^a^		
		Unadjusted		Adjusted	Unadjusted		Adjusted	Unadjusted		Adjusted
	*N*	% ^b^	RRR (95% CI) ^c^	RRR (95% CI) ^c^	% ^b^	RRR (95% CI) ^c^	RRR (95% CI) ^c^	% ^b^	RRR (95% CI) ^c^	RRR (95% CI) ^c^
***Place of residence***										
**Region**			*P* < 0.001	*P* < 0.001		*P* < 0.001	P = 0.021		*P* < 0.001	*P* < 0.001
Phnom Penh	1002	8.1	1	1	1.7	1	1	2.6	1	1
Kratie	1079	14.7	2.18 (1.64–2.90)	1.64 (1.2–2.24)	4.7	3.34 (1.91-5.83)	2.31 (1.27–4.20)	7.4	3.42 (2.18–5.38)	2.66 (1.64–4.32)
Ratanakiri	983	8.3	1.16 (0.84–1.59)	0.76 (0.53–1.09)	5.7	3.76 (2.17–6.53)	2.04 (1.11–3.74)	7.7	3.34 (2.12–5.26)	2.04 (1.24–3.36)
***Socio-economic position***										
**Wealth index**			*P* < 0.001	*P* < 0.001		*P* < 0.001	*P* = 0.061		*P* < 0.001	*P* = 0.119
Poor	1025	13.8	1	1	6.3	1	1	8.7	1	1
Middle	1122	11.5	0.74 (0.57–0.96)	0.82 (0.62–1.09)	3.4	0.47 (0.31–0.72)	0.67 (0.43–1.04)	4.9	0.50 (0.35–0.71)	0.69 (0.48–1.00)
Rich	917	5.7	0.33 (0.24–0.47)	0.41 (0.28–0.59)	2.3	0.29 (0.18––0.48)	0.55 (0.31–0.96)	4.1	0.39 (0.26–0.57)	0.72 (0.46–1.13)
**Mother education**			*P* = 0.096	*P* = 0.878		*P* < 0.001	*P* = 0.095		*P* < 0.001	*P* = 0.156
No formal schooling	1025	11.3	1	1	6.5	1	1	8.7	1	1
Primary	1122	11.1	0.92 (0.68–1.23)	0.92 (0.67–1.27)	3.9	0.55 (0.36–0.83)	0.73 (0.46–1.16)	5.9	0.62 (0.44–0.89)	0.8 (0.55–1.18)
Secondary +	917	9.4	0.73 (0.54–0.99)	0.93 (0.66–1.31)	2.7	0.36 (0.22–0.57)	0.56 (0.33–0.95)	4.2	0.43 (0.29–0.63)	0.65 (0.42–1.01)
***Physiological factors***										
**Gender**			*P* < 0.001	*P* < 0.001		*P* < 0.001	*P* < 0.001		*P* < 0.001	*P* < 0.001
Female	1507	7.1	1	1	6.0	1	1	7.6	1	1
Male	1557	13.8	1.93 (1.51–2.47)	1.93 (1.50–2.48)	2.1	0.35 (0.23–0.52)	0.31 (0.20–0.47)	4.4	0.57 (0.42–0.78)	0.53 (0.39–0.73)
**Age in months**	3064	-	*P* < 0.001	*P* = 0.007	-	*P* < 0.001	*P* < 0.001	-	*P* = 0.496	*P* = 0.016
			1.04 (1.02–1.06)	1.03 (1.01–1.05)		0.91 (0.88–0.94)	0.88 (0.84–0.91)		0.99 (0.96–1.02)	0.96 (0.94–0.99)
**Stunting**			*P* < 0.001	*P* < 0.001		*P* < 0.001	*P* < 0.001		*P* < 0.001	*P* < 0.001
No	2368	9.1	1	1	2.7	1	1	4.1	1	1
Yes	696	15.2	2.19 (1.7–2.82)	1.93 (1.48–2.53)	8.6	4.18 (2.90–6.04)	4.9 (3.28–7.32)	12.1	3.82 (2.81–5.21)	3.78 (2.72–5.26)

^a^ Vs. being a non wasted subject; ^b^ Prevalence proportion; ^c^ RRR = Relative Risk Ratio (95% CI).
